# Changes in retinal and choroidal thickness after carotid endarterectomy: a systematic review

**DOI:** 10.1186/s40942-025-00713-1

**Published:** 2025-08-04

**Authors:** Ana Cibrão-Pedroso, João Rocha-Neves, Rafael Vieira, João Barbosa Breda, André Ferreira

**Affiliations:** 1 Faculty of Medicine, University of Porto, Alameda Professor Hernâni Monteiro, Porto, 4200-319 Portugal; 2 Department of Ophthalmology, Unidade Local de Saúde de Santo António, Porto, Portugal; 3 Department of Biomedicine - Unit of Anatomy, Faculty of Medicine, University of Porto, Porto, Portugal; 4 RISE-Health, Department of Biomedicine, Faculty of Medicine, University of Porto, Alameda Prof. Hernâni Monteiro, Porto, 4200 − 319 Portugal; 5 RISE-Health, Department of Surgery and Physiology, Faculty of Medicine, University of Porto, Porto, Portugal; 6 Department of Ophthalmology, Centro Hospitalar e Universitário São João, Porto, Portugal; 7 KULeuven, Research Group Ophthalmology, Department of Neurosciences, Leuven, Belgium

**Keywords:** Carotid stenosis, Retina, Optical coherence tomography, Postoperative changes, Ophthalmic manifestations

## Abstract

**Background:**

Carotid endarterectomy is a well-established procedure for enhancing cerebral perfusion in patients with internal carotid artery stenosis. As a multifactorial disease, carotid stenosis can have ocular implications, potentially affecting retinal and choroidal perfusion and contributing to visual dysfunction. This systematic review aims to evaluate changes in choroidal and retinal thickness after unilateral carotid endarterectomy, providing insight into the impact of the procedure on ocular perfusion.

**Methods:**

A comprehensive search was performed across PubMed, Scopus, and Web of Science up to July 2024, without restrictions on language or publication date. The inclusion criteria included original studies assessing retinal or choroidal thickness via optical coherence tomography before and after carotid endarterectomy in adults. Additional manual searches of reference lists and citation tracking were employed to ensure completeness. Study quality was appraised via the NHLBI tool for observational studies.

**Results:**

Six prospective observational studies involving 269 patients were included. Findings on choroidal thickness changes after carotid endarterectomy are heterogeneous. While two studies reported significant postoperative Choroidal Thickness increases—one within a week and another at three months—other studies reported no significant changes. One study suggested that higher degrees of carotid stenosis may blunt early Choroidal Thickness response. Retinal measurements were less consistently assessed; among the three studies that evaluated retinal nerve fibre layer and ganglion cell complex thickness, no consistent postoperative changes were observed. Overall, variability in study designs, Optical Coherence Tomography protocols, and follow-up durations limits comparability, precluding meta-analysis.

**Conclusions:**

This review highlights a potential association between carotid endarterectomy and improved ocular perfusion, as reflected by changes in choroidal thickness. However, inconsistencies across studies and limited data on retinal structural outcomes underscore the complexity of this relationship. These findings emphasize the need for larger, standardized studies to clarify the impact of carotid revascularization on the ocular microvasculature and guide future clinical practice.

**Supplementary Information:**

The online version contains supplementary material available at 10.1186/s40942-025-00713-1.

## Background

Carotid artery stenosis (CAS) is a significant vascular condition with far-reaching implications for ocular health, stemming from the intricate relationship between carotid artery circulation and the ocular blood supply [1, 2]. The ophthalmic artery (OA), the first intradural branch of the internal carotid artery (ICA), serves as a critical conduit for ocular microcirculation [3, 4]. OA gives rise to essential vascular structures, including the posterior ciliary arteries and the central retinal artery, which are vital for maintaining retinal and choroidal blood flow [5]. As a result, hemodynamic alterations in the carotid artery may lead to impaired ocular vascularization, potentially resulting in ischemic complications that affect visual function [6, 7].

Carotid endarterectomy (CEA) is a well-established and effective surgical approach for managing moderate- to high-grade CAS in both symptomatic and asymptomatic patients [8, 9]. However, the optimal management of asymptomatic CAS remains a subject of ongoing debate, as advancements in the best medical treatment have raised questions regarding the added benefit of CEA or carotid artery stenting [10, 11]. CEA, although effective in reducing stroke risk in patients with significant carotid artery stenosis, has been occasionally associated with ocular complications. These include retinal artery occlusion, anterior ischemic optic neuropathy (AION), ophthalmic artery occlusion, and transient visual disturbances such as amaurosis fugax. These events are thought to result from embolic phenomena, intraoperative hypoperfusion, or postoperative hemodynamic changes. Although rare, reperfusion injury has also been implicated in postoperative visual loss [12–15].

Optical coherence tomography (OCT) has emerged as a valuable tool for assessing retinal and choroidal structures with high resolution, allowing for detailed analysis of thickness changes that may occur as a result of altered ocular blood flow [16].

CAS has been associated with decreased vessel density in the radial peripapillary capillaries and thinning of the retina and choroid. While recent research has focused on the impact of carotid endarterectomy on these ocular structures, its precise effects remain unclear [2, 17].

This systematic review aims to synthesize existing evidence on variations in retinal and choroidal thickness (CT) before and after CEA, assessed by OCT, in the ipsilateral and contralateral eyes of patients with CAS. By consolidating the current findings, this study contributes to a deeper understanding of ocular changes associated with CAS and CEA, providing a foundation for future research.

## Methods

This systematic review was conducted following the Preferred Reporting Items for Systematic Reviews and Meta-Analyses (PRISMA) 2020 Statement and the AMSTAR-2 (A MeaSurement Tool to Assess Systematic Reviews) critical appraisal tool [18, 19]. The review protocol was prospectively registered in the PROSPERO database (International Prospective Register of Systematic Reviews) with the registration number CRD42024551657.

### Selection criteria

The inclusion criteria consisted of all original articles involving as population: patients who have undergone carotid endarterectomy; intervention: carotid endarterectomy; comparison: preoperative retinal and choroidal thickness measurements; outcomes: changes in retinal and choroidal thickness at various postoperative intervals. Eligible study designs included randomized controlled trials (RCTs), nonrandomized interventional studies, cohort studies, case‒control studies and cases with 20 or more patients. No exclusion criteria based on publication language were applied. Studies focusing solely on other vascular interventions or those not reporting pre- and postoperative OCT measurements were excluded.

### Search strategy

A comprehensive systematic search was conducted in three major electronic databases—PubMed, Scopus, and Web of Science—from inception until July 2024 via a combination of controlled vocabulary (MeSH terms in PubMed) and free-text terms. The detailed search strategy, including all keywords and Boolean operators used, is presented in Supplemental Table 1. We also employed additional search methods to ensure thorough literature coverage. The references of the included primary studies and relevant systematic reviews were manually searched to identify additional articles of interest that may have been missed by the electronic database search. A forward citation search was performed on key articles to find more recent studies that cited them. The search was not restricted by language or publication date to maximize the number of potentially relevant studies retrieved. All the search results were imported into reference management software for deduplication and further screening.

### Study selection and data extraction

After duplicate removal, two authors (ACP and JRN) independently participated in the selection process for the study. Disagreements were resolved by the intervention of a third author (AF). Studies were initially selected based on title and abstract, and the remaining studies were eligible for full-text assessment. Full texts that were not publicly available were obtained by contacting the authors or relevant organizations. The selected studies were carefully reviewed to avoid repeated populations. Data from the included studies were independently extracted by two authors (ACP and JRN) using a purposely built form. The data extracted from the primary studies included country, publication year, recruitment period, study center, and study design. Further extracted variables included choroidal thickness, retinal nerve fibre layer thickness, and ganglion cell layer thickness before and after CEA. Additionally, data on participant demographics and baseline characteristics, including the number of participants and controls (when applicable), sex, mean age, number of eyes, number of carotid interventions, mean carotid stenosis, presence of symptoms, use of single or double antiplatelet therapy and presence of postsurgery complications such as ischemic optic neuropathy, stroke, death and hematoma, were collected. This review specifically focuses on structural parameters, such as retinal and choroidal thickness measured by conventional optical coherence tomography (OCT).

### Assessment of study quality

The methodological quality of the included studies was evaluated using the National Heart, Lung, and Blood Institute (NHLBI) Study Quality Assessment Tool for observational cohort and cross-sectional studies (2021) [20]. This assessment was independently performed by two authors (ACP and JRN). Any disagreements were resolved through discussion and consensus, with a third author (AF) consulted when necessary. The risk of bias in the included studies was visualized via the Robvis tool, which allows for clear representation across key domains [21].

## Results

### Search results

**Fig. 1:**
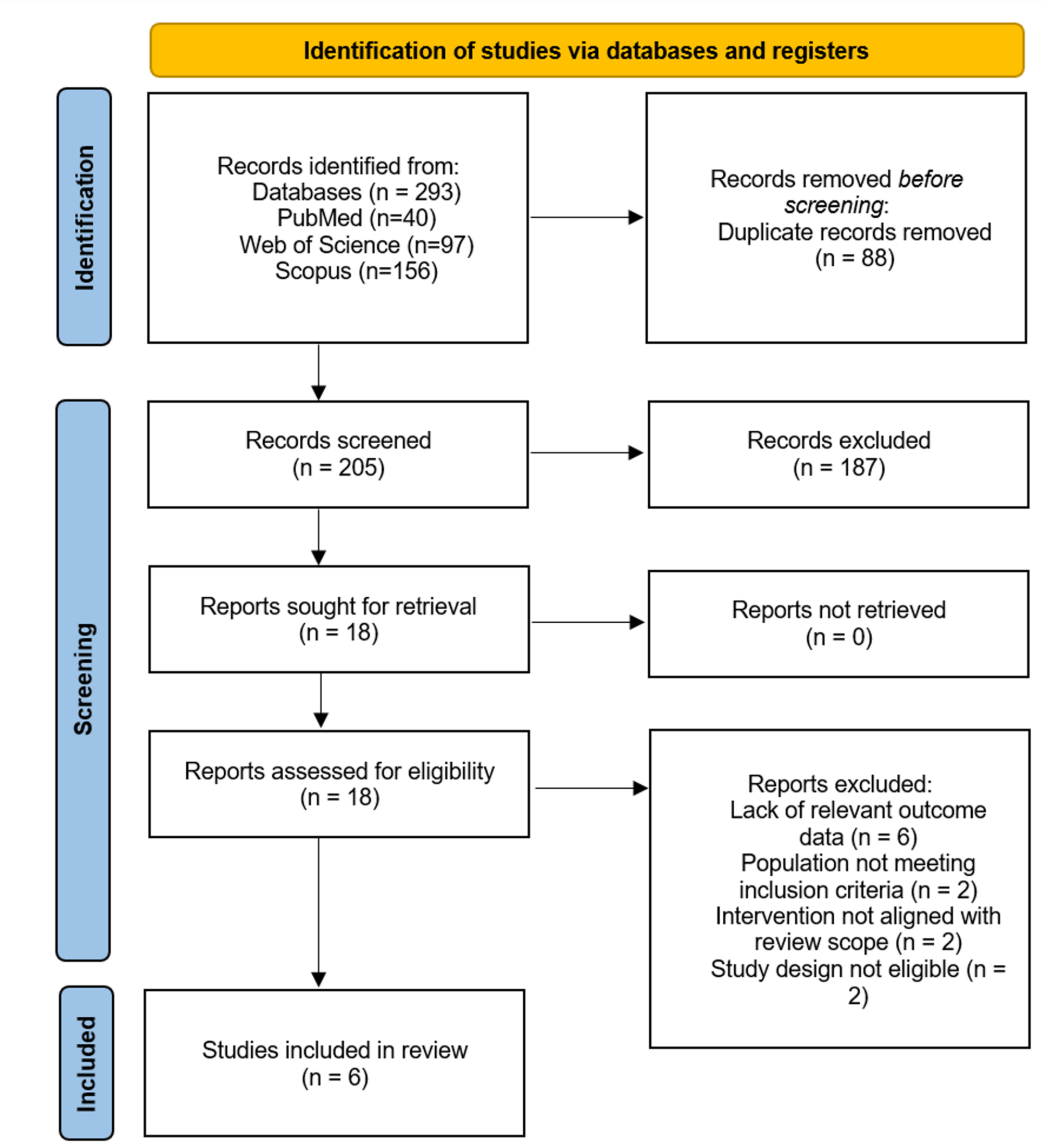
PRISMA flow diagram showing the selection of included articles for review

A systematic literature search was conducted across three databases. After removing duplicates, 205 unique records were identified for screening. Title and abstract screening excluded 187 records, leaving 18 full-text articles to be assessed for eligibility. Of these, 12 studies were excluded for the following reasons: lack of relevant outcome data (*n* = 6), population not meeting inclusion criteria (*n* = 2), intervention not aligned with the review scope (*n* = 2) and study design not eligible (*n* = 2). All the eligible full-text articles were successfully retrieved. Ultimately, 6 studies met the inclusion criteria and were included in the systematic review. The study selection process is presented in Fig. 1, adhering to the PRISMA 2020 flow diagram.

### Description of the studies

This systematic review included six studies [22–27] published between 2017 [25] and 2024 [27], employing prospective observational study designs. The studies were conducted across six countries spanning three continents: Israel [27], Turkey [23], Finland [22], Poland [24, 25], and Italy [26]. A total of 269 patients were assessed across all studies, with individual study sample sizes ranging from 21 [24] to 70 [22] participants. The main characteristics of the included studies are summarized in Table 1. The study populations were predominantly middle-aged to elderly individuals, with mean ages ranging from 63.5 [25] to 71.57 [27] years. The sex distribution varied considerably, with male participation ranging from 48% [24] to 83% [27] across studies. A detailed overview of the demographic and comorbidity profiles of each study population is provided in Table 2, while key participant characteristics, including carotid stenosis severity, symptoms, and perioperative management, are summarized in Table 3.

**Table 1:** Main characteristics of the included studies

Author Year Country	Study Design	Study Center	Recruitment Period	Sample Size (*n*)	No CEA
**Zhang et al. (27)** **2024** Israel	Prospective observational	Tel Aviv Medical Center	NR	60	60
**Guclu et al. (23)** **2018** Turkey	Prospective observational	Trakya University	NR	42	42
**Ala-Kauhaluoma et al. (22)** **2020** **Finland**	Prospective observational	Helsinki University Hospital	March 2015 - December 2018	70	70
**Krytkowska et al. (24)** **2020** Poland	Prospective observational	Pomeranian Medical University	NR	21	21
**Pierro et al. (26)** 2021 Italy	Prospective observational	San Raffaele Hospital	January 2018 - September 2019	30	30
**Machalińska et al.(25)** 2017 Poland	Prospective observational	Pomeranian Medical University	NR	46	46

Abbreviations: NR, not reported; CEA, carotid endarterectomy

**Table 2:** Participants’ demographic and clinical characteristics

Author, Year	No patients (no eyes)	Age (years), mean ± SD	Male	AHT	Dyslipidemia	Statins	DM	BMI (mean)	Smoking History	CAD	PAD	HF
Zhang et al., 2024 (27)	60 (116)	71.6 ± 7.4	50 (83%)	NR	NR	NR	NR	NR	NR	NR	NR	NR
Guclu et al., 2018 (23)	42 (84)	63.7 ± 5.6	30 (71%)	21 (50%)	21	NR	11 (26%)	NR	3 (7%)	NR	NR	NR
Ala-Kauhaluoma et al., 2020 (22)	70 (140)	69 ± 7	57 (81%)	58 (83%)	66 (94%)	57 (81%)	24 (34%)	27.7	23 (33%)	22 (32%)	NR	NR
Krytkowska et al., 2020 (24)	21 (42)	66.4 ± 8.2	10 (48%)	11 (52%)	NR	NR	NR	27.3	16 (76%)	6 (31.6%)	NR	NR
Pierro et al., 2021 (26)	30 (60)	68 ± 8	17 (57%)	30 (100%)	NR	NR	12 (40%)	NR	NR	NR	NR	NR
Machalińska et al., 2017 (25)	46 (92)	63.5 ± 6.1	27 (59%)	38 (83%)	NR	NR	NR	27.8	42 (91%)	16 (35%)	12 (26%)	NR

Abbreviations: NR, not reported; SD, standard deviation; AHT, arterial hypertension; BMI, body mass index; CAD, coronary artery disease; PAD, peripheral artery disease; HF, heart failure

**Table 3:** Characteristics of the included Participants– Carotid stenosis severity, symptoms, and perioperative management

Author, Year	Carotid Stenosis Mean (%)		Amaurosis Fugax	Symptomatic Carotid Stenosis	SAPT	DAPT	Anticoagulation^1^	Anaesthesia Type
	Ipsilateral	Contralateral						
Zhang et al., 2024 (27)	84.2%	48.1%	NR	NR	NR	NR	NR	General
Guclu et al., 2018 (23)	> 70%	NR	NR	NR	NR	NR	NR	General
Ala-Kauhaluoma et al., 2020 (22)	< 50% in 4% 50–69% in 4% 70–99% in 42% Subtle near occlusion in 40% Near occlusion with full collapse in 9% Total occlusion in 1%	< 50% in 74% 50–69% in 7% 70–99% in 10% Subtle near occlusion in 3% Near occlusion with full collapse in 1% Total occlusion in 4%	25 (36%)	NR	94%		NR	NR
Krytkowska et al., 2020 (24)	81.5%	29.5%	NR	NR	100%^2^		100%	Regional
Pierro et al., 2021 (26)	NR	NR	NR	5 (17%)	Yes, but not specified		100%	Regional
Machalińska et al., 2017 (25)	77%	12%	0	0	100%^2^		100%	Regional

Abbreviations: NR, not reported; SAPT, single antiplatelet therapy; DAPT, dual antiplatelet therapy

^1^Systemic heparinization during surgery

^2^The patients were preoperatively treated with oral acetylsalicylic acid at a dosage of 75 mg/d for at least 10 days prior to CEA

### Main findings

The findings revealed a complex and varied picture across the included studies. Notably, only three studies specifically assessed retinal parameters. Tables 4 and 5 provide a detailed summary of the OCT measurements recorded before and after CEA.

**Table 4:** Optical coherence tomography measurements before and after carotid endarterectomy—choroidal thickness (CT)

Author, Year	OCT machine	Timing of postoperative OCT	MCT^1^ (µm)			
			Before CEA		After CEA	
			EIE	ECE	EIE	ECE
Zhang et al., 2024 (27)	LEX Elite 9000, Carl Zeiss Meditec	1 week	245.0 ± 99.7	257.7 ± 96.3	**264.8 ± 99.1** **(** ***p*** **< 0.001)**	258.8 ± 93.3
Guclu et al., 2018 (23)	RS-3000 Lite, Nidek, Japan	1 month	NR	NR	NR	NR
Ala-Kauhaluoma et al., 2020 (22)	Spectralis, Heidelberg Engineering, Germany	6 months	222 ± 54	217 ± 60	225 ± 61	209 ± 54
Krytkowska et al., 2020 (24)	Spectralis, Heidelberg Engineering, Germany	2 days; 3 months;	299 ± 72	289 ± 72	291 ± 77; **310 ± 77** **(** ***p*** **= 0.04)**	272 ± 65; **295 ± 65** **(** ***p*** **= 0.02)**
Pierro et al., 2021 (26)	DRI OCT Topcon Triton, Japan	3 months	223.3 ± 67.4	285.4 ± 65.7	224.4 ± 68.8	302 ± 67.0
Machalińska et al., 2017 (25)	NR	3 months	NR	NR	NR	NR

Abbreviations: EIE, eyes ipsilateral to endarterectomy; ECE, eyes contralateral to endarterectomy; OCT, optical coherence tomography; CEA, carotid endarterectomy; MCT, mean choroidal thickness; RNFL, retinal nerve fibre layer; NR, not reported; NA, not applicable

Statistically significant results are indicated in bold

^1^ Mean choroidal thickness is reported for all studies but Ala-Kauhaluoma et al., who presented only subfoveal measurements

**Table 5:** Optical coherence tomography measurements before and after carotid endarterectomy– retinal nerve fibre layer thickness

Author, Year	OCT machine	Timing of postoperative OCT	RNFL Thickness^1^ (µm)															
			Before CEA								After CEA							
			EIE				ECE				EIE				ECE			
			S	*N*	I	T	S	*N*	I	T	S	*N*	I	T	S	*N*	I	T
Zhang et al., 2024 (27)	LEX Elite 9000, Carl Zeiss Medi- tec	1 week	NR	NR	NR	NR	NR	NR	NR	NR	NR	NR	NR	NR	NR	NR	NR	NR
Guclu et al., 2018 (23)	RS-3000 Lite, Nidek, Japan	1 month	121 (107–140)	86 (77–101)	119 (98–132)	70 (58–79)	114 (100–127)	81 (53–88)	130 (105–152)	67 (60–95)	**110 (98–330)** *p* = 0.04	80 (75–94)	121 (98–142)	64 (53–72)	122 (106–132)	75 (55–97)	125 (104–140)	63 (53–65)
Ala-Kauhaluoma et al., 2020 (22)	Spectralis, Heidelberg Engineering, Germany	6 months	NR	NR	NR	NR	NR	NR	NR	NR	NR	NR	NR	NR	NR	NR	NR	NR
Krytkowska et al., 2020 (24)	Spectralis, Heidelberg Engineering, Germany	2 days; 3 months;	NR	NR	NR	NR	NR	NR	NR	NR	NR	NR	NR	NR	NR	NR	NR	NR
Pierro et al., 2021 (26)	DRI OCT Topcon Triton, Japan	3 months	102.6 ± 10,3				105.5 ± 10.3				NR				NR			
Machalińska et al., 2017 (25)	NR	3 months	91.4 ± 8,0				91.4 ± 9.8				91.6 ± 8.7				92.6 ± 8.7			

Abbreviations: EIE– Eyes Ipsilateral to Endarterectomy; ECE– Eyes Contralateral to Endarterectomy; OCT - Optical Coherence Tomography; CEA - Carotid Endarterectomy; Subfoveal CT - Subfoveal Choroidal Thickness; RNFL - Retinal Nerve Fibre Layer; NR - Not Reported; NA - Not Applicable

Statistically significant results are indicated in bold

^1^Guclu et al. presented RNFL thickness as the median (^25th–75th^ percentiles)

Four studies evaluated baseline Choroidal Thickness (CT) in both ipsilateral and contralateral eyes prior to CEA, with inconsistent results. Zhang et al. [27] reported thinner CT in the ipsilateral eye than in the contralateral eye. Ala-Kauhaluoma et al. [22] and Krytkowska et al. [24] reported no significant interocular differences, although Ala-Kauhaluoma et al. [22] also noted that both eyes presented thinner CTs than healthy controls did. Similarly, Pierro et al. [26] reported thinner CT only in the ipsilateral eye than in controls.

Postoperative changes in choroidal thickness have demonstrated inconsistent findings across studies. Zhang et al. [27] reported a significant increase in CT on the ipsilateral side, observing this change within one week postoperatively. In contrast, Ala-Kauhaluoma et al. [22] and Pierro et al. [26] reported no significant postoperative increase in CT, whereas Krytkowska et al. [24] reported a significant decrease 3 months after surgery. Interestingly, Krytkowska et al. [24] also reported a significant increase in CT in the contralateral eye at three months after surgery. Furthermore, they noted that patients with more severe internal carotid artery stenosis were less likely to exhibit a CT increase within the first two days following CEA, suggesting that the degree of stenosis may influence early postoperative choroidal responses. Notably, all the studies reported the mean choroidal thickness except for Ala-Kauhaluoma et al. [22], who presented only subfoveal measurements.

Findings on retinal nerve fibre layer (RNFL) and ganglion cell complex (GCC) thickness after CEA were mixed. Guclu et al. [23] reported significant thinning of the superior peripapillary RNFL in the ipsilateral eye but found no significant changes in GCC thickness. In contrast, Pierro et al. [26] and Machalińska et al. [25] reported no significant changes in RNFL or GCC thickness in either eye.

Table 6 summarizes the postoperative complications reported in the included studies, with most studies either not reporting data or documenting no occurrences. The only exception was Ala-Kauhaluoma et al. [22], who reported one case of stroke.

**Table 6:** Postoperative complications reported in included studies

Author, Year	Ischemic Optic Neuropathy	Stroke	Death	Hematoma
Zhang et al., 2024 (27)	NR	NR	NR	NR
Guclu et al., 2018 (23)	NR	NR	NR	NR
Ala-Kauhaluoma et al., 2020 (22)	NR	1	NR	NR
Krytkowska et al., 2020 (24)	NR	0	0	NR
Pierro et al., 2021 (26)	0	NR	0	NR
Machalińska et al., 2017 (25)	NR	0	NR	NR

Abbreviations: NR– Not reported

Owing to the limited number of studies and methodological differences, a quantitative analysis was not feasible, accentuating the need for more standardized research approaches in future studies.

### Study quality

**Fig. 2:**
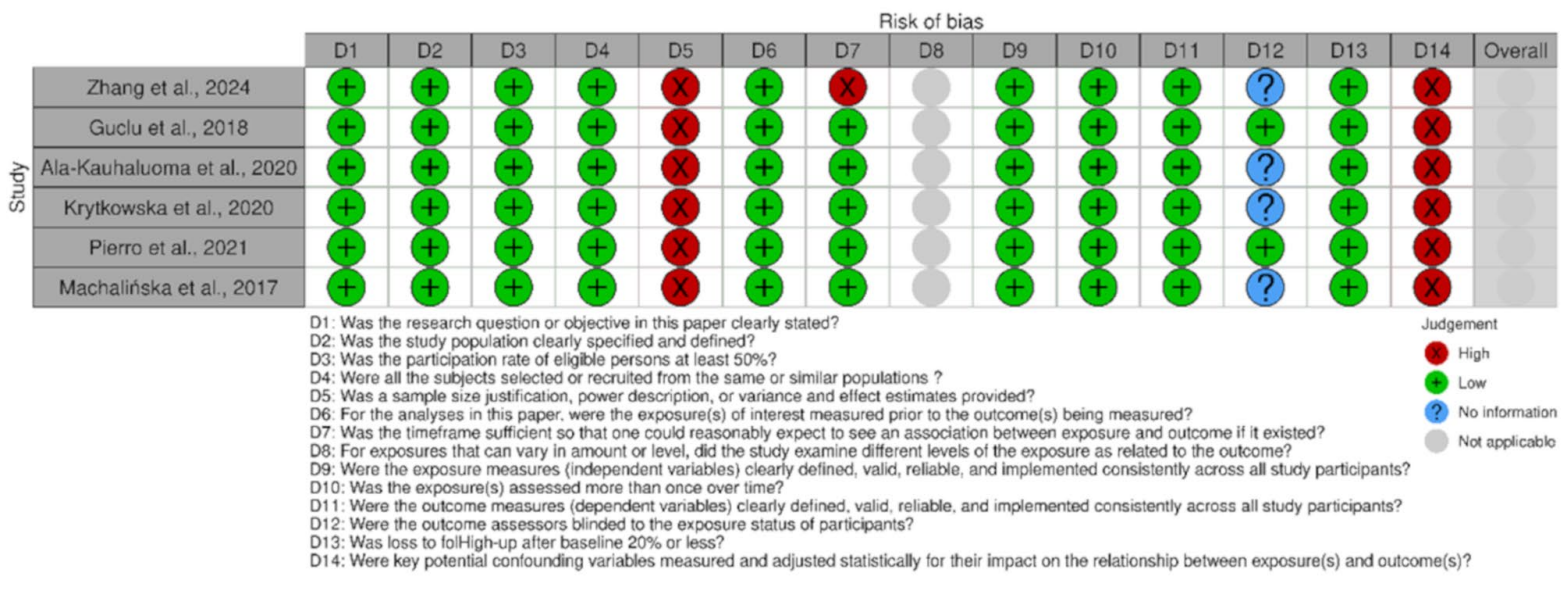
Traffic Light Plot - Risk of bias assessment generated with the Robvis tool

**Fig. 3:**
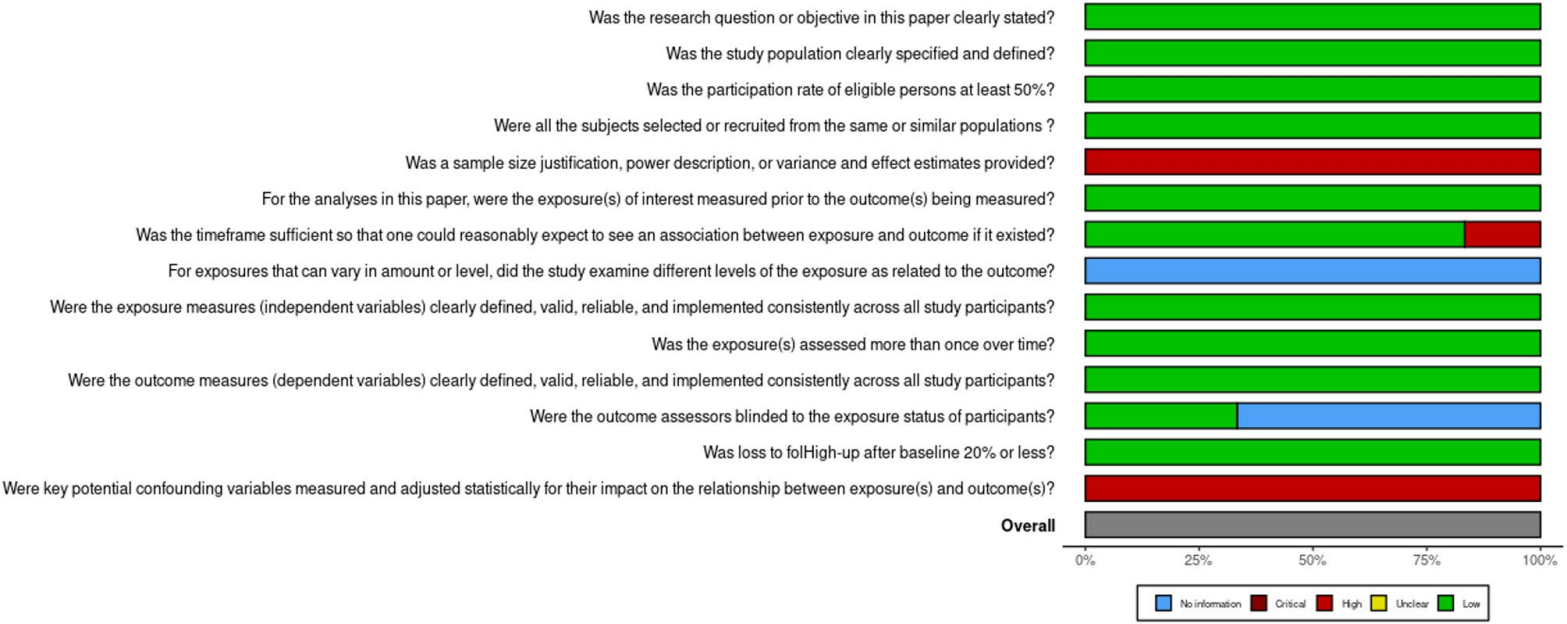
Summary plot- Risk of bias assessment generated with the Robvis tool

The risk of bias assessment, as illustrated in Figs. 2 and 3, revealed several methodological limitations across the included studies. A consistent area of concern was the lack of sample size justification, power description, or variance and effect estimates, which were rated as high risk for all studies. Additionally, inadequate adjustment for potential confounding variables was another critical source of bias, with all studies exhibiting a high risk in this domain. While most studies clearly stated their research question/objective, specified the study population, had participation rates above 50%, and recruited subjects from similar populations, these strengths can be overshadowed by limitations in statistical rigor and confounding control. Furthermore, the blinding of the outcome assessors was often unclear. Notably, interpreting these results requires caution, suggesting areas for improvement in future research.

## Discussion

This systematic review synthesized existing evidence on retinal and choroidal thickness variations following CEA, as assessed via OCT. Some of the included studies demonstrated significant postoperative changes in retinal and choroidal thickness, reporting increased thickness after surgery. However, variations in patient characteristics and measurement protocols contributed to the heterogeneity in the results, highlighting the need for standardized methodologies in future research.

The six included studies, which were conducted across multiple countries, mainly employed prospective observational designs to evaluate retinal and choroidal changes in patients undergoing CEA. Sample sizes varied (21–70 patients), and recruitment strategies also differed: two studies incorporated control groups [22, 26], whereas the remaining studies exclusively examined post-CEA outcomes [24, 25, 27].

To avoid duplication of data, particular attention was paid to studies conducted by the same research group or at the same site. Although two included studies by Krytkowska et al. (2020) [24] and Machalińska et al. (2017) [25] were conducted at the same institution by overlapping authors, they reported on distinct outcome parameters, choroidal thickness and retinal function respectively, and thus were both included, as they contributed non-overlapping data.

The study population predominantly consisted of male patients, except for the study by Krytkowska et al. [24], which reported a more balanced sex distribution. These populations often present high rates of cardiovascular risk factors, such as arterial hypertension and smoking habits, along with frequent systemic comorbidities. These factors underscore the complex link between systemic vascular health and ocular microvascular changes, highlighting the need to consider potential confounders.

Most patients presented severe stenosis, typically exceeding 70%, ipsilaterally. The inconsistent reporting of preoperative ocular symptoms points to variability in retinal ischemic manifestations. However, overt ischemic retinal changes were assessed by fundoscopy in all patients, with two studies reporting ocular ischemic syndrome as exclusion criteria. Moreover, differences in perioperative management strategies, including antithrombotic therapy and anaesthesia type, may play a significant role in shaping postoperative ocular outcomes. These findings call for further research to explore how these variables influence ocular circulation and postoperative recovery.

There were notable inconsistencies in the baseline CT measurements across the included studies. Two of the six studies reported CT in the eyes ipsilateral to the CEA compared with the contralateral eyes [26, 27]. Specifically, Zhang et al. [27] associated the choroidal thinning observed in the ipsilateral eye with reduced OA perfusion caused by stenosis of the carotid artery. Similarly, Pierro et al. [26] reported significantly thinner CT in ipsilateral eyes than in control eyes, further supporting the notion of compromised baseline choroidal perfusion in these patients. These findings align with previous research suggesting impaired choroidal circulation, which is attributed to decreased perfusion of the posterior ciliary arteries as a result of diminished ophthalmic artery perfusion in severe internal carotid artery stenosis [28–30]. On the other hand, two studies reported no significant difference in baseline CT between the ipsilateral and contralateral eyes [22, 24], which aligns with the results of Rabina et al. [31]. These conflicting results may be attributed to preserved OA perfusion despite carotid artery stenosis [31], variations in disease severity or the presence of compensatory mechanisms that maintain choroidal blood flow. In line with the findings of Akcay et al. [1], Krytkowska et al. [24] suggested that in cases of severe ICA stenosis, CT may increase, likely resulting from choriocapillaris vasculature dilation. This response appears to be a protective adaptation to mitigate retinal and choroidal ischemia due to the reduced blood flow caused by ICA stenosis. These varying findings suggest that while decreased baseline choroidal thickness in stenotic patients’ eyes is consistent with impaired ocular perfusion, the presence of compensatory mechanisms and variable preservation of ophthalmic artery perfusion may account for the heterogeneity observed across studies. Notably, choroidal thickness can be influenced by several physiological and external variables, which may affect the accuracy and interpretability of measurements. Key factors include age, axial length, refractive error, diurnal variation, systemic blood pressure, and hydration status. Additionally, factors such as smoking, caffeine intake, and the use of certain systemic medications may also impact choroidal thickness [32]. Only one study [22] prohibited caffeine intake and smoking before the exams. Additionally, the choroidal thickness measurement varied between studies, with two using the built-in software of the OCT machine [24, 27] and the other two measuring it manually [22, 26]. The choroidal vascular index (CVI) has been proposed as a more robust choroidal parameter and has already been assessed in several ocular and systemic diseases [33]. As advantages, this parameter appears to be more predictable and is less affected by other variables than CT is [34]. Berni et al. [35] assessed the CVI after carotid endarterectomy and reported no differences after the procedure.

Regarding postoperative CT, two of the included studies reported a significant increase in choroidal thickness in the ipsilateral eyes following CEA [24, 27]. Zhang et al. [27] reported a marked increase in CT within one week post-CEA, which aligns with previous research showing an increase in CT following both carotid stenting [36] and CEA [37]. This increase suggests an enhancement in ocular perfusion following surgical intervention. However, Ala-Kauhaluoma et al. [22] did not observe significant changes in CT postoperatively, suggesting that long-term ischemia in patients with severe carotid stenosis might hinder the choroid’s ability to fully adapt. Similarly, Krytkowska et al. [24] reported no early postoperative changes in CT but observed significant improvements at three months postoperatively, suggesting that vascular autoregulation mechanisms might temporarily prevent an immediate increase in choroidal perfusion after CEA, with more noticeable changes occurring over the longer term. However, choroidal blood flow regulation remains a debated topic, particularly because, unlike the retina, the choroid exhibits only limited autoregulatory capacity [38–40]. While retinal circulation is known for its robust intrinsic autoregulatory mechanisms, the choroid relies predominantly on extrinsic control, especially from the autonomic nervous system [41, 42]. Krytkowska et al. [24] also reported CT increase in the contralateral eyes, postoperatively, with statistical significance reached at three months. This finding suggests a positive effect on both cerebral and ocular perfusion via the circle of Willis and ophthalmic arteries, a phenomenon commonly referred to as “redistribution” [24, 28]. The variability in postoperative CT changes may be attributed to differences in carotid stenosis severity, preoperative ocular symptoms, perioperative management strategies and imaging techniques, all of which could influence the extent of choroidal recovery after CEA.

The impact of CEA on RNFL and GCC thickness warrants careful consideration, as the literature presents inconsistent results. Guclu et al. [23] reported significant thinning of the peripapillary RNFL in the superior quadrant of ipsilateral eyes one month after CEA, suggesting a potential vulnerability of specific RNFL regions to ischemia‒reperfusion injury associated with the procedure. The authors hypothesized that ischaemia‒reperfusion injury may reduce Vascular Endothelial Growth Factor (VEGF) levels and that the relatively short period of ischemia could have caused localized superior peripapillary RNFL defects in the ipsilateral eye. In contrast, Machalińska et al. [25] and Pierro et al. [26] did not report significant changes in RNFL thickness within three months following CEA. Furthermore, none of the three studies, Guclu et al. [23], Machalińska et al. [25], or Pierro et al. [26], reported significant changes in GCC thickness in either the ipsilateral or contralateral eyes during the same postoperative period. It is possible that the inner retinal layers are not sufficiently sensitive to changes induced by carotid artery stenting (CAS) or carotid endarterectomy (CEA). Therefore, it would be relevant to include data regarding the whole retina, particularly given that the outer retinal layers are supplied primarily by the choroidal circulation. Furthermore, the retina may exhibit limited responsiveness to CAS/CEA compared with the choroid, which is a highly vascularized structure with minimal autoregulatory capacity.

This systematic review, while providing valuable insights into choroidal and retinal thickness changes following CEA, is subject to several limitations that should be considered when interpreting its findings. First, the limited number of eligible studies and small sample sizes reduce the statistical power of the review. The absence of sample size justifications and power analyses in the primary studies further contributes to the low precision of the results. This limitation increases the risk of Type I and Type II errors, making it difficult to conclusively determine the true effect size of CEA on choroidal thickness. Second, there is considerable variability across the studies in terms of patient characteristics and methodologies. Differences in choroidal measurements and follow-up durations introduce challenges in making direct comparisons and rendering a meta-analysis unfeasible. Furthermore, variations in patient selection criteria, such as the presence of preoperative ocular symptoms and the severity of carotid stenosis, may further account for the observed heterogeneity. Additionally, the relatively short follow-up periods in several studies raise concerns regarding the sustainability of the observed outcomes. Th resource to different OCT devices across studies may introduce some variability, particularly due to differences in image acquisition protocols and resolution. However, this potential source of heterogeneity is mitigated by the fact that each individual study employed a single OCT device consistently across all participants, ensuring reliable intra-study comparisons. Furthermore, our analyses are based on relative differences rather than absolute measurements, which helps to minimize the impact of inter-device variability on the overall findings. Finally, the limited number of studies reporting functional visual outcomes makes it difficult to assess whether the observed structural changes in choroidal thickness correlate with clinical improvements. Although choroidal thickness assessments provide valuable anatomical insights, their clinical significance remains unclear without corresponding functional data. Optical coherence tomography angiography (OCTA), which offers detailed visualization of the retinal and choroidal microvasculature, could enhance the understanding of the vascular impact of CEA beyond structural measurements alone and could be a more valuable tool in this setting. To address these limitations, larger, high-quality cohort studies are needed to confirm the findings and reduce bias. Studies assessing long-term retinal and choroidal changes post-CEA, as well as investigations into the role of preoperative carotid stenosis severity and systemic vascular risk factors, would provide valuable insights. Finally, standardization of OCT measurement protocols is crucial to improve study comparability.

## Conclusions

This systematic review provides a comprehensive overview of the current evidence regarding retinal and choroidal thickness changes following carotid endarterectomy. While most studies suggest the potential for improved ocular perfusion, as evidenced by increased choroidal thickness after CEA, inconsistencies in baseline measurements and postoperative changes highlight the complex interplay of factors influencing ocular microvasculature in the context of carotid artery disease. The mixed findings regarding the retinal nerve fibre layer and ganglion cell complex thickness highlight the need for a more nuanced understanding of the impact of CEA on various retinal structures.

The significant heterogeneity in patient characteristics and methodologies underscores the challenges in drawing definitive conclusions from the existing literature, with small sample sizes, a lack of standardized OCT protocols and limited reporting of functional visual outcomes, further restricting the generalizability of the findings. These limitations highlight the need for larger, well-designed, and standardized studies to validate the observed trends and better elucidate the long-term effects of CEA on ocular health.

In conclusion, this review demonstrates the potential association between carotid revascularization and improvements in ocular perfusion while also identifying key areas for future research. Ultimately, a better understanding of the relationship between carotid artery disease and the ocular microvasculature will pave the way for improved patient selection, monitoring and optimization of therapeutic approaches.

## Electronic Supplementary Material

Below is the link to the electronic supplementary material.

Supplementary Material 1

## Data Availability

No datasets were generated or analysed during the current study.

## Abbreviations

CAS: Carotid artery stenosis
OA: Ophthalmic artery
ICA: Internal carotid artery
CEA: Carotid endarterectomy
AION: Anterior ischemic optic neuropathy
OCT: Optical coherence tomography
PRISMA: Preferred Reporting Items for Systematic Reviews and Meta-Analyses
AMSTAR-2: A MeaSurement Tool to Assess Systematic Reviews
PROSPERO: International Prospective Register of Systematic Reviews
RCTs: Randomized controlled trials
OCTA: Optical coherence tomography angiography
NHLBI: National Heart, Lung, and Blood Institute
CT: Choroidal thickness
RNFL: Retinal nerve fibre layer
GCC: Ganglion cell complex
CVI: Choroidal vascular index
SAPT: Single antiplatelet therapy
DAPT: Dual antiplatelet therapy
VEGF: Vascular endothelial growth factor
NR: Not reported
SD: Standard deviation
BMI: Body mass index
CAD: Coronary artery disease
PAD: Peripheral artery disease
HF: Heart failure
EIE: Eyes ipsilateral to endarterectomy
ECE: Eyes contralateral to endarterectomy
