# Supplementary material for: Changes in retinal and choroidal thickness after carotid endarterectomy: a systematic review
Source: Int J Retina Vitreous. 2025 Aug 4;11:91. doi: 10.1186/s40942-025-00713-1 (PMC12323285; doi:10.1186/s40942-025-00713-1)
Supplement: Supplementary file 1 — Supplementary Material 1 [file 40942_2025_713_MOESM1_ESM.docx]

**Supplementary Table 1** – Search terms and queries.

| Bibliographic source | Search term | N° of reports |
| --- | --- | --- |
| Pubmed | ((("tomography, optical coherence"[MeSH Terms] OR ("tomography"[All Fields] AND "optical"[All Fields] AND "coherence"[All Fields]) OR "optical coherence tomography"[All Fields] OR ("optical"[All Fields] AND "coherence"[All Fields] AND "tomography"[All Fields])) AND ("Endarterectomy" OR "Endarterectomy, Carotid" OR “carotid endarterectomy” OR “Carotid Endarterectomies”)) | 40 at 26/07/2024 |
| Web of Science | TS= (( "Optical Coherence Tomography " OR "OCT”) AND TS= ("Endarterectomy" OR "Endarterectomy, Carotid" OR “carotid endarterectomy” OR “Carotid Endarterectomies”) | 97 at 26/07/2024 |
| Scopus | TITLE-ABS-KEY(("Optical Coherence Tomography" OR "OCT") AND ("Endarterectomy" OR "Endarterectomy, Carotid" OR “carotid endarterectomy” OR “Carotid Endarterectomies”)) | 156 at 26/07/2024 |
